# Identification and function analysis of yellow-leaf mutant (*YX-yl*) of broomcorn millet

**DOI:** 10.1186/s12870-022-03843-y

**Published:** 2022-09-27

**Authors:** Yushen Wang, Junjie Wang, Liqing Chen, Xiaowei Meng, Xiaoxi Zhen, Yinpei Liang, Yuanhuai Han, Hongying Li, Bin Zhang

**Affiliations:** 1 College of Agriculture, Shanxi Agricultural University, Taigu, Shanxi China 030801; 2 Shanxi Key Laboratory of Germplasm Innovation and Molecular Breeding of Minor Crop, Taigu, Shanxi China 030801; 3 Ministerial and Provincial Co-Innovation Centre for Endemic Crops Production With High-Quality and Efficiency in Loess Plateau, Shanxi Agricultural University, Taigu, Shanxi China 030801; 4 Institute of Agricultural Bioengineering, Shanxi Agricultural University, Taigu, Shanxi China 030801

**Keywords:** Broomcorn millet, *YX-yl* mutant, Chlorophyll synthesis, Chloroplast structure, Transcriptome sequencing

## Abstract

**Background:**

Broomcorn millet is highly tolerant to drought and barren soil. Changes in chlorophyll content directly affect leaf color, which subsequently leadsleading to poor photosynthetic performance and reduced crop yield. Herein, we isolated a yellow leaf mutant (*YX-yl*) using a forward genetics approach and evaluated its agronomic traits, photosynthetic pigment content, chloroplast ultrastructure, and chlorophyll precursors. Furthermore, the molecular mechanism of yellowing was explored using transcriptome sequencing.

**Results:**

The *YX-yl* mutant showed significantly decreased plant height and low yield. The leaves exhibited a yellow-green phenotype and poor photosynthetic capacity during the entire growth period. The content of chlorophyll a, chlorophyll b, and carotenoids in *YX-yl* leaves was lower than that in wild-type leaves. Chlorophyll precursor analysis results showed that chlorophyll biosynthesis in *YX-yl* was hindered by the conversion of porphobilinogen to protoporphyrin IX. Examination of chloroplast ultrastructure in the leaves revealed that the chloroplasts of *YX-yl* accumulated on one side of the cell. Moreover, the chloroplast structure of *YX-yl* was degraded. The inner and outer membranes of the chloroplasts could not be distinguished well. The numbers of grana and grana thylakoids in the chloroplasts were low. The transcriptome of the yellowing mutant *YX-yl* was sequenced and compared with that of the wild type. Nine chlorophyll-related genes with significantly different expression profiles were identified: *PmUROD*, *PmCPO*, *PmGSAM*, *PmPBDG*, *PmLHCP*, *PmCAO*, *PmVDE*, *PmGluTR*, and *PmPNPT*. The proteins encoded by these genes were located in the chloroplast, chloroplast membrane, chloroplast thylakoid membrane, and chloroplast matrix and were mainly involved in chlorophyll biosynthesis and redox-related enzyme regulation.

**Conclusions:**

*YX-yl* is an ideal material for studying pigment metabolism mechanisms. Changes in the expression patterns of some genes between *YX-yl* and the wild type led to differences in chloroplast structures and enzyme activities in the chlorophyll biosynthesis pathway, ultimately resulting in a yellowing phenotype in the *YX-yl* mutant. Our findings provide an insight to the molecular mechanisms of leaf color formation and chloroplast development in broomcorn millet.

**Supplementary Information:**

The online version contains supplementary material available at 10.1186/s12870-022-03843-y.

## Background

The leaves are the main organs for photosynthesis and are vital for plant growth and development. Leaf traits, especially leaf color, are considered important morphological traits that are relevant to photosynthesis potential and grain yield. Leaf color is mainly determined by the content of chlorophyll and carotenoids, which are photosynthetic pigments. In higher plants, photosynthetic pigments fix light energy and convert it into chemical energy to synthesize carbohydrates. Chlorophyll is the primary photosynthetic pigment that harvests light and drives electron transport in reaction centers [1]. Leaf color mutants with photosynthetic pigment deficiency or abnormal chloroplast structures can serve as a subject for systematic investigation of the mechanisms of photosynthesis and photosynthetic pigment biosynthesis or degradation in plants [2].

Recently, leaf-color mutants of different crops or plants, such as rice [3–6], wheat [7], foxtail millet [8], *Arabidopsis thaliana* [9], and Chinese cabbage [10], have been used to identify steps associated with chlorophyll metabolism or chloroplast development. Studies have elucidated the molecular mechanisms of leaf-color mutants, especially the yellowing phenotype, which is most commonly observed in higher plants. Yellow-leaf mutants usually have shorter plant height, lower grain yield, deficiency in photosynthetic pigments, and abnormal chloroplast ultramicrostructures compared with the wild type [7, 11–14]. For example, the yellowish-leaf mutant *ydl* of rice has photosynthetic pigment deficiencies, degraded chloroplasts, and disordered matrix [4]. Genes that regulate leaf color formation and chloroplast development have been identified in numerous plants [2, 4, 5, 8–11, 13]. More than 70 genes corresponding to leaf color variations have been cloned [15]. Mutations in the *BC12/GDD1* gene resulted in low chlorophyll content and short plant height in rice, and a mutation in the EMBRYO DEFECTIVE (EMB1923) gene promoter is associated with chlorophyll deficiency in Chinese cabbage [4, 10]. A novel gene *SiYGL1* in foxtail millet is responsible for a deficiency in chlorophyll content [8]. An incomplete dominant gene *Y1718* in a yellow-green leaf mutant (*Ygm*) contributed to yellow, yellow-green, and green leaf phenotypes from the jointing to adult stage [7]. Virus-induced gene silencing of the *Mg chelatase CHL1* and *CHLD* (*CHL1* and *CHLD)* genes both resulted in a yellow leaf phenotype in pea [16]. A single base mutation in the *AtCHL1* gene led to a yellow-green or white phenotype in *Arabidopsis* [17].

Moreover, both internal and external factors, such as temperature, salt, and biotic and abiotic stresses, can influence photosynthetic pigment content and chloroplast development through alterations in gene expression or post-translational modification of proteins involved in chlorophyll metabolism [3, 4, 18–21]. For example, in common vegetable crops, photosynthetic pigment metabolism and chloroplast development are disrupted under drought stress [22]. An *Arabidopsis* mutant deficient in *stay-green2* (*SGR2*) exhibited early leaf yellowing under age-, darkness-, and stress-induced senescence conditions [9]. Chloroplast development is also controlled by several hormones, including brassinosteroids, cytokinins, auxins, and gibberellins, which control chloroplast development, particularly during the early stages of plant development [23]. Several BR-related mutants in *Arabidopsis* have fewer grana stacks than the wild type [24–26]. In addition, CTK promotes several steps in chlorophyll biosynthesis, including the formation of 5-aminolevulinic acid (ALA; the first step in the biosynthesis pathway of tetrapyrroles) and light-dependent conversion of protochlorophyllide (Pchlide) into chlorophyllide [27–29]. In *Arabidopsis*, 29 chloroplast-related proteins, including CAB (LHCB1), LSU, and LHCB2, respond to auxin treatment [30]. Overexpression of *AUXIN RESPONSE FACTOR 10* (*SlARF10*) and *SlARF6A* results in increased chlorophyll content and photosynthesis rate in the leaves, whereas *SlARF10*-RNAi and *SlARF6A* knockdown lines have less chlorophyll content than the wild type [31, 32]. Moreover, gibberellin affects the development of the cellular chloroplast compartment by controlling both cell expansion and cell division [33]. Deficiency in gibberellin biosynthesis results in a low number of cells and small cell size in *Arabidopsis* and rice plants, which leads to a decrease in chloroplast division and total chloroplast number at the whole-leaf level [34].

**Fig. 1:**
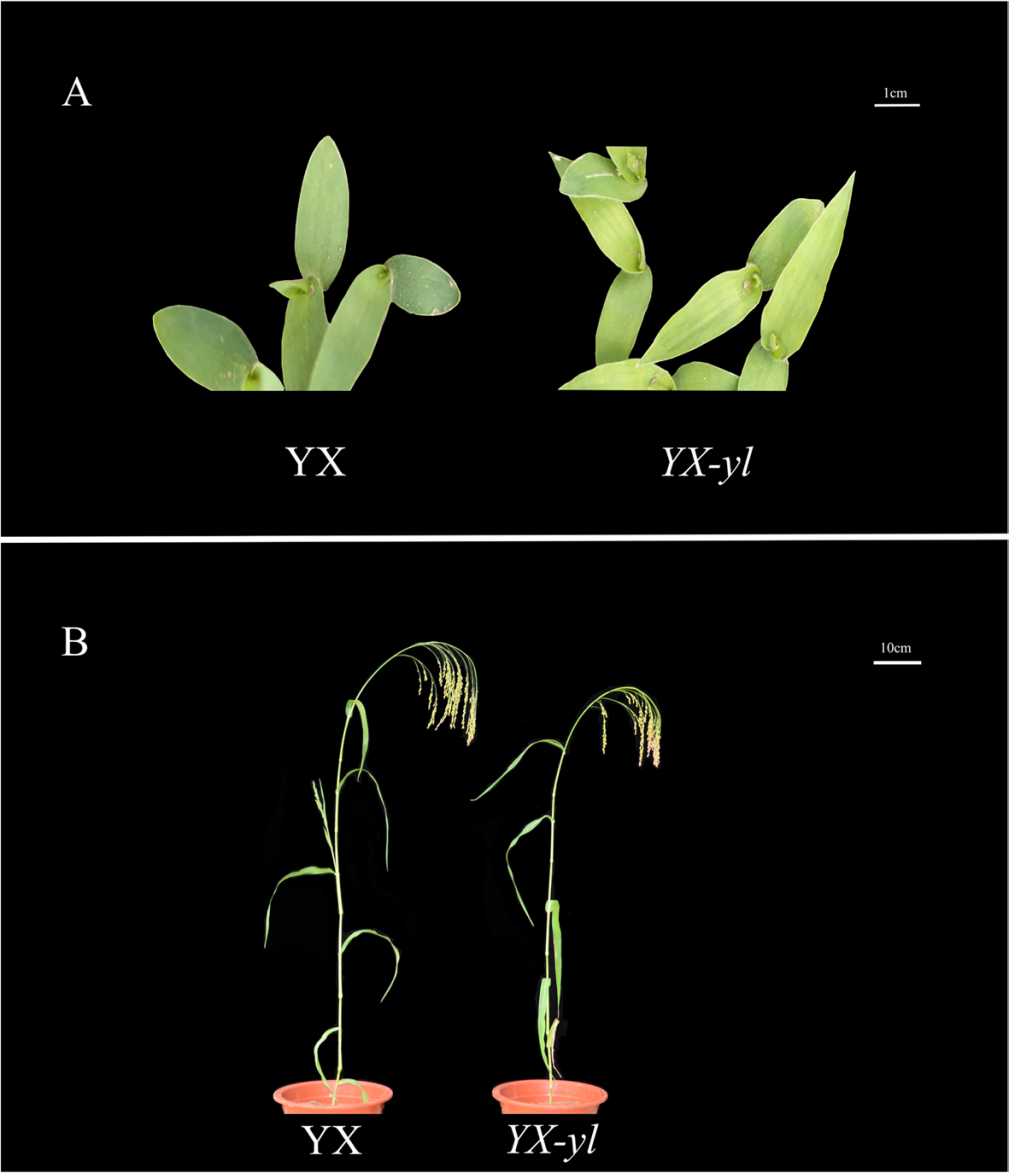
Phenotype characterization of yellow-leaf mutant *YX-yl* and wild-type YX. *YX-yl* and YX at the (**A**) seedling stage (scale bar: 5 cm) and (**B**) harvesting stage (scale bar: 10 cm)

Broomcorn millet (*Panicum miliaceum* L.), one of the world’s oldest cultivated crops, has excellent water use efficiency and is mainly used for dryland farming, where most other crops fail to survive [35]. Before the domestication of rice and wheat, broomcorn millet was the major food in many semiarid regions of Asia, including China, Korea, India, Russia, Japan, and even the entire Eurasian continent [36]. Broomcorn millet performs C_4_ photosynthesis, which is more efficient than C_3_ photosynthesis. Much effort has been made to engineer C_4_ traits in C_3_ crops, such as rice. This requires a clear understanding of the molecular mechanisms underlying C_4_ chlorophyll metabolism and photosynthesis. Currently, a yellow-leaf phenotype mutant (*YX-yl*) is isolated from the Yi Xuan Da Hong Mei cultivar (YX) mutant library. *YX-yl* exhibited a yellow phenotype throughout the growth stage (Fig. 1). However, the molecular mechanisms underlying this phenotype remain unclear.

In the present study, the agronomic traits, photosynthetic pigments and characteristics, water use efficiency, chlorophyll fluorescence dynamic parameters, chloroplast ultrastructure, intermediate metabolites, and transcriptome-level changes in *YX-yl* and YX were analyzed. Based on phenotypic, physiological, and bioinformatic analyses, we identified nine genes related to chlorophyll biosynthesis and chloroplast development. Our results illustrate the mechanism of leaf yellowing in broomcorn millet and provide insights into the molecular mechanisms underlying chlorophyll metabolism and chloroplast development.

## Results

### Phenotypic performance of *YX-yl* mutant

Compared with those of the wild type (YX), the plant height (PH), spike weight per plant (SWPP), and grain weight per plant (GWPP) of *YX-yl* were significantly decreased by 21.4%, 81.1%, and 79.5%, respectively (*P* < 0.01). Additionally, the flag leaf width (FLW), basal stem diameter (BSD), and peduncle length (PL) of *YX-yl* were significantly shorter than those of YX, showing decreases of 29.1%, 31.3%, and 30.5%, respectively (Table 1).

**Table 1:** Comparison of major agronomic traits between the *YX-yl* mutant and YX wild-type

Agronomic traits	YX	*YX-yl*	Reduction rate/%
Plant height/cm	167.12 ± 2.417	131.36 ± 2.048**	-21.40%
Stem diameter/mm	6.56 ± 0.087	4.51 ± 0.473*	-31.30%
Peduncle length/mm	34.79 ± 1.578	24.18 ± 1.607*	-30.50%
Flag leaf length/cm	38.06 ± 0.91	31.57 ± 1.325	-17.10%
Flag leaf width/cm	2.34 ± 0.118	1.66 ± 0.107*	-29.10%
Panicle length/cm	33.18 ± 0.330	36.51 ± 1.618	10.00%
Spike weight per plant/g	6.5 ± 0.052	1.23 ± 0.053**	-81.10%
Grain weight per plant/g	5.33 ± 0.035	1.09 ± 0.066**	-79.50%

Data are presented as the mean ± SD based on three individuals. Asterisks indicate a significant difference between YX and *YX-y l*: *n* = 3, Welch’s two-sample *t*-test, *P < 0.05, **p < 0.01

### Chlorophyll biosynthesis analysis

**Fig. 2:**
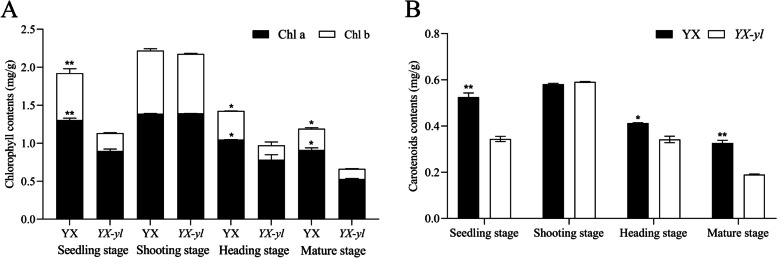
Comparison of pigment (chlorophyll and carotenoid) content in the yellow-leaf mutant *YX-yl* and wild-type YX. The content of chlorophyll a (Chl a) (**A**), chlorophyll b (Chl b) (**A**), and carotenoid (Car) (**B**) in the leaves were measured. Error bars represent ± SD. * and ** indicate significant differences (*p* < 0.05, *p* < 0.01)

Assessment of chlorophyll and carotenoid content in *YX-yl* and YX leaves at different growth stages showed that the yellow phenotype was probably due to deficiency in photosynthetic pigments (Fig. 2 and Table S1). Compared with that of YX, the total chlorophyll content of *YX-yl* was significantly reduced by 41.0%, 31.6%, and 44.5% at the seedling, heading, and mature stages, respectively. Upon further analysis, the Chl b content showed a greater decreasing trend than the Chl a content during the entire growing stage (Fig. 2A). The Chl b content of *YX-yl* significantly decreased by 60.6%, 50.0%, and 53.6% at the seedling, heading, and mature stages, respectively (*p* < 0.05), whereas the Chl a content of *YX-yl* was reduced by 31.3% (seedling stage), 25.7% (heading stage), and 41.8% (mature stage) compared to that of YX.

The stability of chlorophyll could be maintained by carotenoids in the chloroplast. Therefore, we investigated the Car content of YX and *YX-yl*. The results showed that the Car content of *YX-yl* significantly decreased at the seedling, heading, and mature stages compared with that of YX (Fig. 2B). All these reductions in chlorophyll and carotenoid levels led to the yellow-leaf phenotype in the *YX-yl* mutant.

**Fig. 3:**
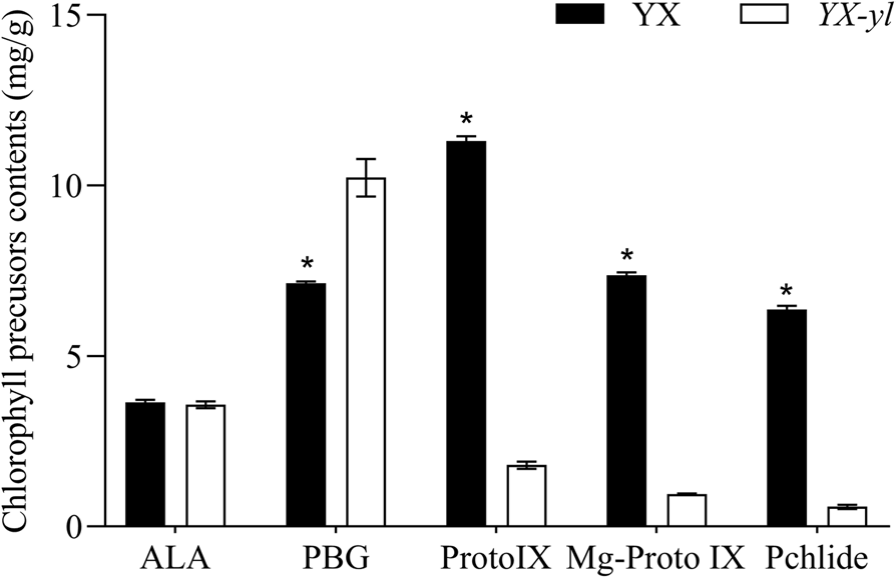
Comparison of the relative content of chlorophyll precursors. Three individuals were measured for each chlorophyll precursor. Error bars represent ± SD. * and ** indicate significant differences (*p* < 0.05, *p* < 0.01). ALA, 5-aminolevulinic acid; PBG, porphobilinogen; Proto IX, protoporphyrin IX; Mg-Proto IX, Mg-protoporphyrin IX; Pchlide, protochlorophyllide

Furthermore, we investigated the biochemical steps of chlorophyll biosynthesis to determine the step whose disruption caused the yellow-leaf phenotype. Five intermediate products related to chlorophyll biosynthesis metabolic processes were compared (Fig. 3). The results showed that protoporphyrin IX (Proto IX), Mg-protoporphyrin IX (Mg-Proto IX), and Pchlide content in the leaves of the *YX-yl* mutant were significantly lower than that in YX leaves, whereas the porphobilinogen (PBG) content of the *YX-yl* mutant was much higher than that of YX plants. The levels of PBG, Proto IX, Mg-Proto IX, and Pchlide in the *YX-yl* mutant were 143.43%, 15.99%, 12.91%, and 9.09% of those in YX, indicating that inhibition of the biosynthesis step between PGB and Proto IX might be the reason for the reduction in the chlorophyll content of *YX-yl*.

### Chloroplast ultrastructure analysis

**Fig. 4:**
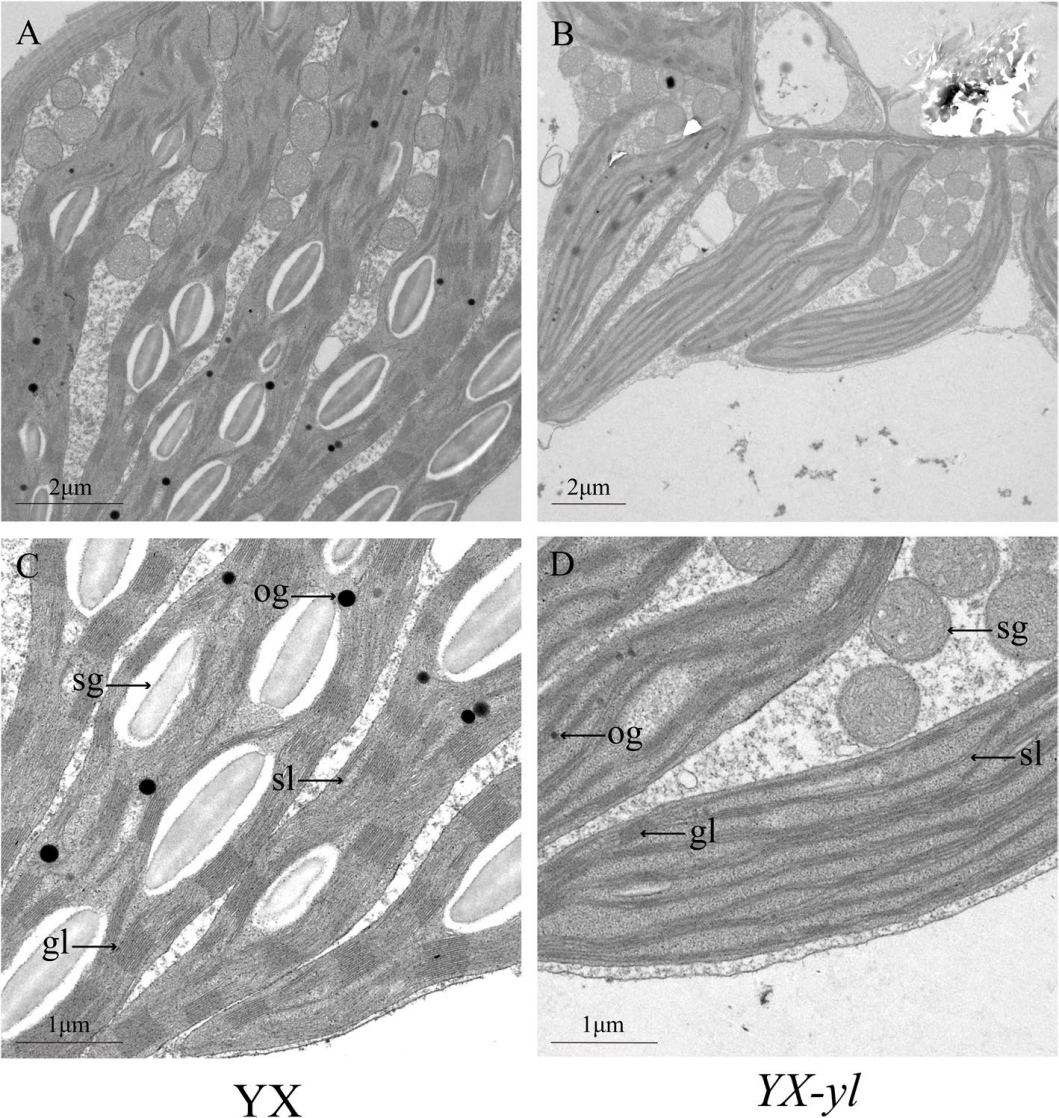
Transmission electron micrograph of chloroplasts from mutant (*YX-yl*) and wild-type (YX) broomcorn millet. Structure of chloroplasts in YX (**A** and **C**) and *YX-yl* (**B** and **D**). sg, starch grains; sl, stroma lamellae; gl, grana lamellae; og, osmiophilic granules. Bar: 2 μm (**A** and **B**) and 1 μm (**C** and **D**)

Chloroplasts are important organelles for photosynthesis in plants; therefore, we assessed the chloroplast ultrastructure of YX and *YX-yl* using transmission electron microscopy (Fig. 4). No differences were found in the number and size of chloroplasts between YX and *YX-yl* plants (Table 2). However, the chloroplast distributions in mesophyll cells were different. Chloroplasts were evenly distributed in the mesophyll cells of YX, but only on one side of the mesophyll cell of the *YX-yl* mutant. Furthermore, the chloroplast ultrastructures of YX and *YX-yl* leaves were assessed. Compared to that in YX, chloroplast development was inhibited in *YX-yl*, and the outer and inner membranes of chloroplasts in *YX-yl* could not be clearly distinguished. A lower number of grana and grana thylakoids and almost no stromal thylakoids were observed in the chloroplasts of *YX-yl* leaves.

**Table 2:** Comparison of chloroplasts between the *YX-yl* mutant and YX wild-type

Characters	YX	*YX-yl*
Length(μm)	5–9	8–14
Width(μm)	3–7	2–5
Number	4.5 ± 0.025	5.4 ± 0.024 ns
Shape	oval or circle	long strip shape

Data are presented as mean ± SD based on 30 replications or a range of values. ns indicates no significant difference between YX and *YX-yl*. Asterisks indicate a significant difference between YX and *YX-yl*: *n* = 30, Welch’s two-sample *t*-test, *P < 0.05, **p < 0.01

### Photosynthetic characterization and fluorescence kinetic parameter assessment

**Fig. 5:**
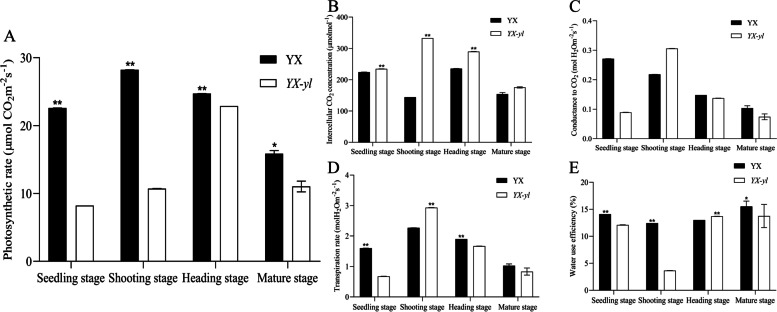
Photosynthetic performance of YX and *YX-yl* broomcorn millet. Each condition includes measurements for three plants. Error bars represent ± SD. * and ** indicate significant differences (*p* < 0.05, *p* < 0.01). Pn; Net photosynthetic rate; Ci, intercellular CO_2_ concentration; Tr, transpiration rate; Gs, stomatal conductance; WUE, water use efficiency

**Fig. 6:**
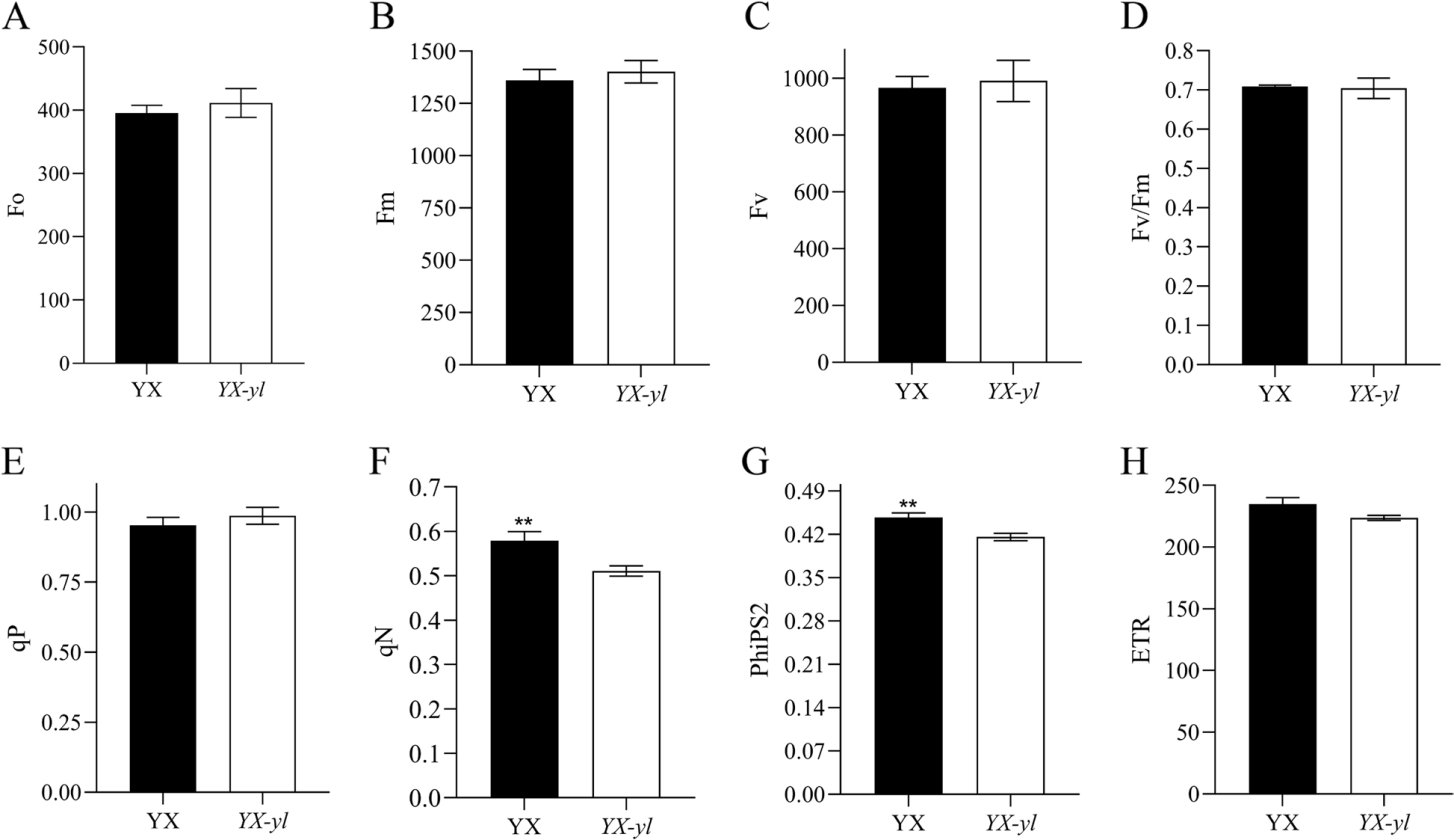
Fluorescence kinetic parameters in YX and *YX-yl*. Each condition includes measurements for three plants. Error bars represent ± SD. * and ** indicate significant differences (*p* < 0.05, *p* < 0.01). PhiPS2, the proportion of light quantum absorbed by PSII that is used for photochemical reactions; qN, non-photochemical quenching coefficient; Fo, minimal fluorescence; Fm,_:_ maximal fluorescence under dark adaptation; Fv/Fm, maximum energy conversion efficiency in PSII centers; qP, photochemical quenching coefficient; ETR, photosynthetic electron transport rate; Fv, variable fluorescence

To investigate whether the reduction in the content of chlorophyll pigments and chloroplast development defects affected photosynthesis in *YX-yl* mutants, we examined photosynthesis-related parameters and chlorophyll fluorescence (Figs. 5 and 6). During the entire growth stage, the net photosynthetic rate (Pn) of the *YX-yl* mutant was even lower than that of YX. The Pn of *YX-yl* was only 36.4%, 38%, 92.6%, and 69.5% of that of YX at the seedling, shoot, heading, and mature stages, respectively. Compared with YX, *YX-yl* showed significant decreases in stomatal conductance to CO_2_ (Gs) and transpiration rate (Tr) during the entire growth stage, except during the shooting stage. We also found that the intercellular CO_2_ concentration (Ci) of *YX-yl* in the four growth stages was higher than that of YX, which indicated that the lower Pn of the *YX-yl* mutant might be partially due to a decrease in the CO_2_ assimilation rate.

Plants with high water use efficiency (WUE) are essential for maintaining growth and grain yield in drought environments. We examined the WUE of YX and *YX-yl* leaves, and the results showed that *YX-yl* had an even lower WUE during the whole growth stage, and the biggest difference was observed in the shooting stage, where the WUE of *YX-yl* was only 29.4% of that of YX (Fig. 5).

Fluorescence kinetic parameters reflect the ability of plants to absorb and transfer light. Eight parameters were investigated to evaluate light transfer efficiency (Fig. 6). A significant reduction was observed in the PhiPS2 of *YX-yl* leaves, which indicated a relatively lower proportion of light absorbed by PSII that was used for photochemical reactions (*p* < 0.01). The non-photochemical quenching coefficient (qN, percentage of PSII efficiency loss due to heat dissipation) was also lower in *YX-yl* plants (*p* < 0.05), indicating a decrease in its photoprotection ability. Other parameters such as minimal fluorescence (Fo, related to chlorophyll concentration in the leaf), maximal fluorescence under dark adaptation (Fm, reflecting the electron transfer through PSII), maximum energy conversion efficiency in PSII centers (Fv/Fm), and photochemical quenching coefficient (qP, suggesting that the proportion of PSII reaction centers that are active) did not significantly change. These results indicate that the reduction in chlorophyll and carotenoid content, as well as the lower qN and PhiPS2, might be the reasons for the low photosynthetic rate and light-use efficiency in the *YX-yl* mutant.

### Transcriptome sequencing and unigene annotation

To explore the molecular mechanism of the yellow-leaf phenotype of the *YX-yl* mutant, cDNA libraries of YX and *YX-yl* plants were constructed based on three biological replicates (Table S2). The total number of paired-end reads in the three replicates of *YX-yl* was 31,285,839, 32,403,426, and 31,314,942, respectively, whereas that in YX was 35,356,911, 33,846,844, and 34,511,262, respectively. Moreover, the three samples of YX and *YX-yl* showed a strong correlation (Fig. S1). Clean reads were obtained by removing adapters, low-quality reads, poly N reads, and empty reads. The final yield was approximately 59.38 G, with a GC percentage ranging from 59.41 to 60.60%. The Q30 values of the raw data were all above 94.96%, indicating a high read-confidence level.

Owing to the lack of reference genomes with high mapped ratios, higher-quality reads were used to construct unigene libraries. The total number of unigenes was 100,737, and the N50 of unigenes was 1071, indicating that unigenes had a high assembly integrity. Sequence alignment was performed between clean and unigene libraries (Table S3). More than 59.23% (59.39 to 62.77%) of clean reads in the three biological replications of YX and *YX-yl* were mapped to the unigene libraries. Moreover, seven databases, including NR, Swiss-Prot, GO, COG, KOG, eggNOG4.5, and KEGG, were used to annotate the unigene libraries using the BLAST software. A total of 63,501 unigenes with annotation information were obtained, accounting for 63.04% of the Unigene database (Table S4).

### Identification of differentially expressed gene (DEGs)

**Fig. 7:**
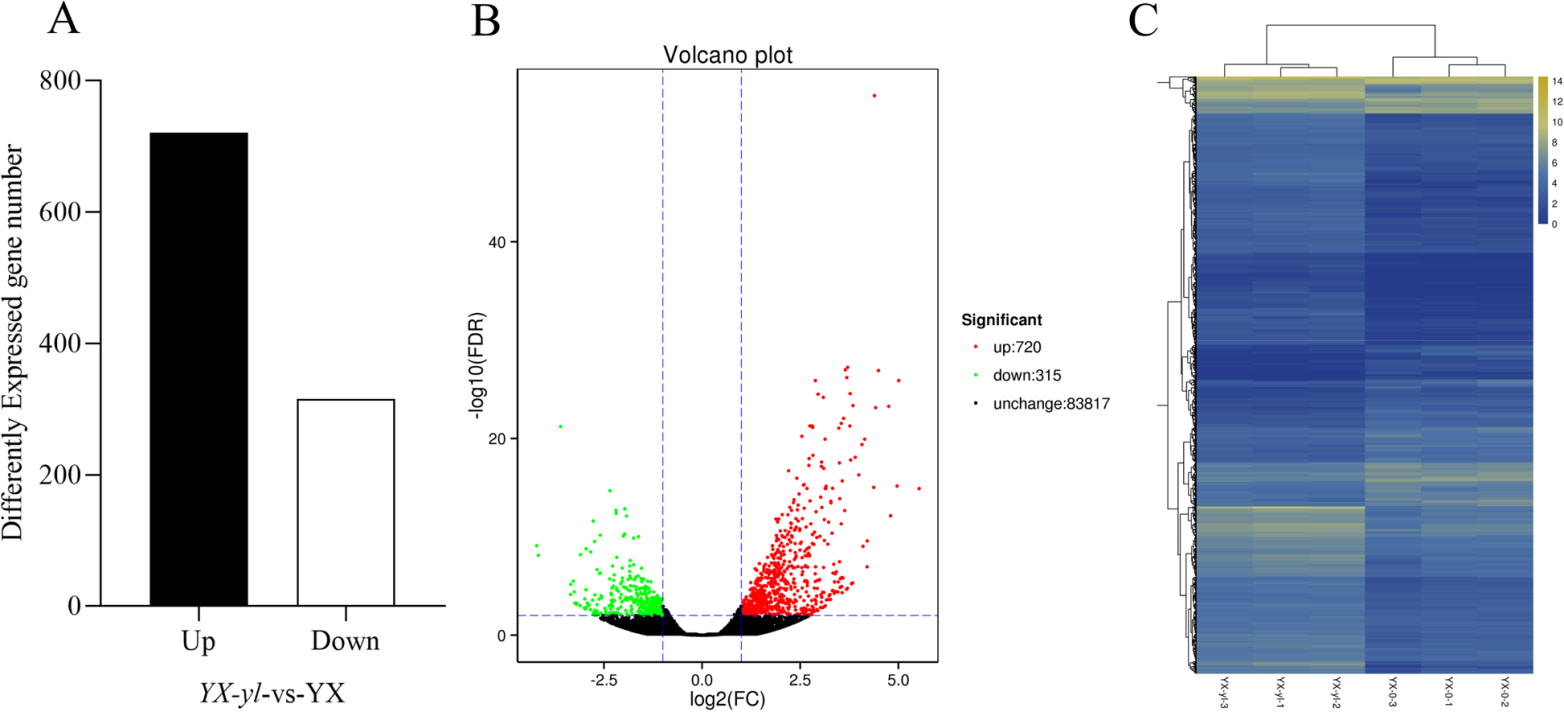
Transcriptome analysis of DEGs in *YX-yl* and YX. **A** Comparison of DEGs in *YX-yl* and YX. **B** Volcano plot showing the DEGs between two different libraries. PDR < 0.01 and FC ≥ 2 were used to determine the significance of DEGs. Red and green dots represent up- and downregulated genes, respectively, and black dots indicate transcripts that did not significantly change in Y*X-yl* compared to that in YX. **C** Hierarchical clustering of all DEGs was based on the FRKM values

A total of 1035 DEGs between *YX-yl* and YX were identified, including 720 upregulated and 315 downregulated genes (Fig. 7A and B). Hierarchical clustering of the DEGs was conducted to assess gene expression patterns. The expression level was calculated using the FPKM of the genes in YX and *YX-yl* (Fig. 7C). Furthermore, 1035 DEGs were mapped with NR, Swiss-Prot, GO, COG, KOG, eggNOG4.5, and KEGG using the BLAST software, and 912 of the 1035 DEGs had annotation information (Table S4).

### GO and KEGG analyses of DEGs

**Fig. 8:**
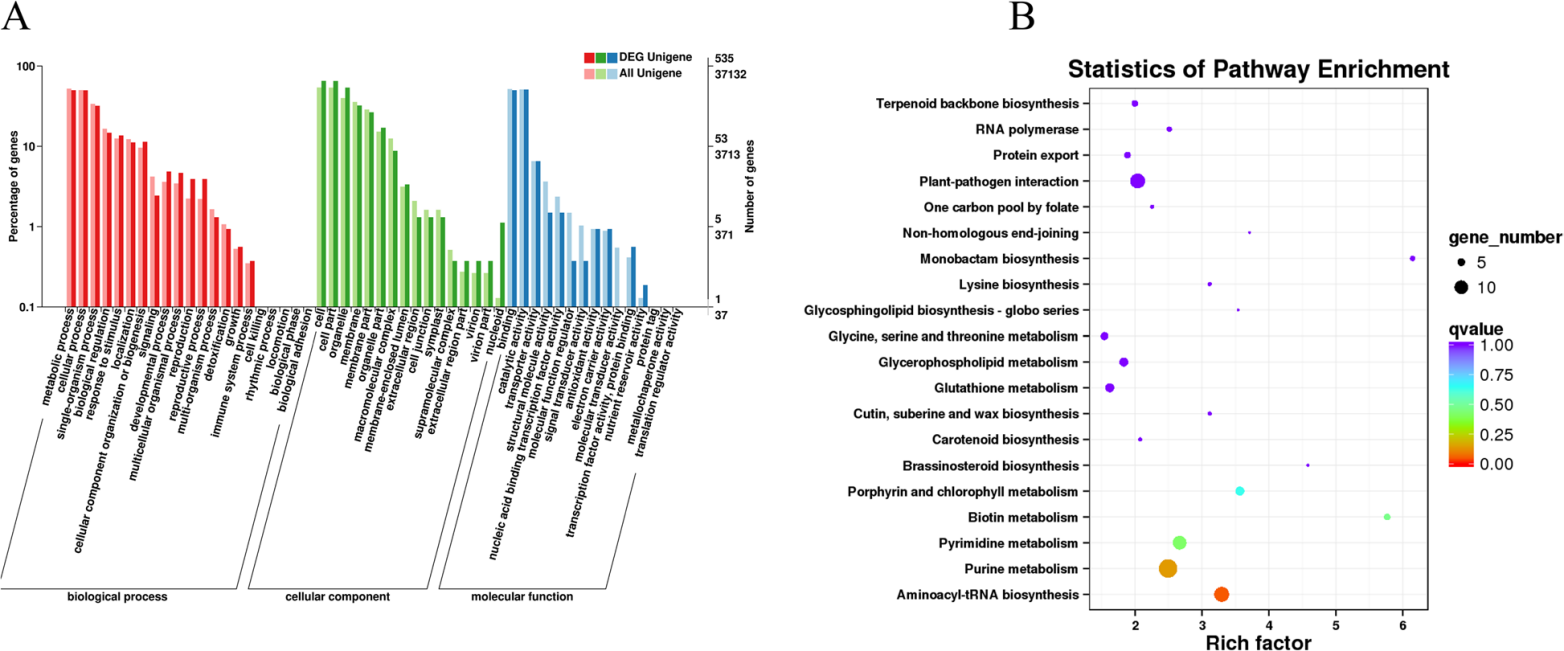
GO and KEGG pathway enrichment analyses of DEGs between YX and *YX-yl*. **A** GO enrichment analysis with 30 most-enriched GO terms in the three categories shown. **B** KEGG enrichment analysis with 20 most-enriched KEGG terms shown

To explore the DEGs involved in leaf etiolation in the *YX-yl* mutant, GO assignments were conducted to classify the functions of the DEGs. In total, 912 DEGs were classified into biological processes, cellular components, and molecular functions. Some DEGs were annotated with more than one GO term, and many DEGs were annotated to biological processes and cellular components (Fig. 8A). In the biological process category, numerous genes belong to “metabolic process (GO:0,008,152),” “cellular process (GO:0,009,987),” and “single-organism process (GO:0,044,699).” Other genes were related to cellular components, namely “cell (GO:0,005,623),” “cell part (GO:0,044,464),” and “intracellular (GO:0,005,622)”. The molecular function category mainly included “binding (GO:0,005,488),” “catalytic activity (GO:0,003,824),” “organic cyclic compound binding (GO:0,057,159),” and “heterocyclic compound binding (GO:1,901,363).”

KEGG pathway analysis was conducted to categorize gene functions, with an emphasis on biochemical pathways that are active in the leaves of *YX-yl* and YX plants (Fig. 8B). In total, 13,704 unigenes and 176 DEGs were annotated and assigned to 93 pathways (Table S5). The most enriched pathways were “aminoacyl-tRNA biosynthesis,” “purine metabolism,” “pyrimidine metabolism,” “biotin metabolism,” and “porphyrin and chlorophyll metabolism.” These results indicate that the yellow-leaf phenotype was mainly due to differences in metabolic processing.

### Identification of DEGs related to chlorophyll metabolism

**Fig. 9:**
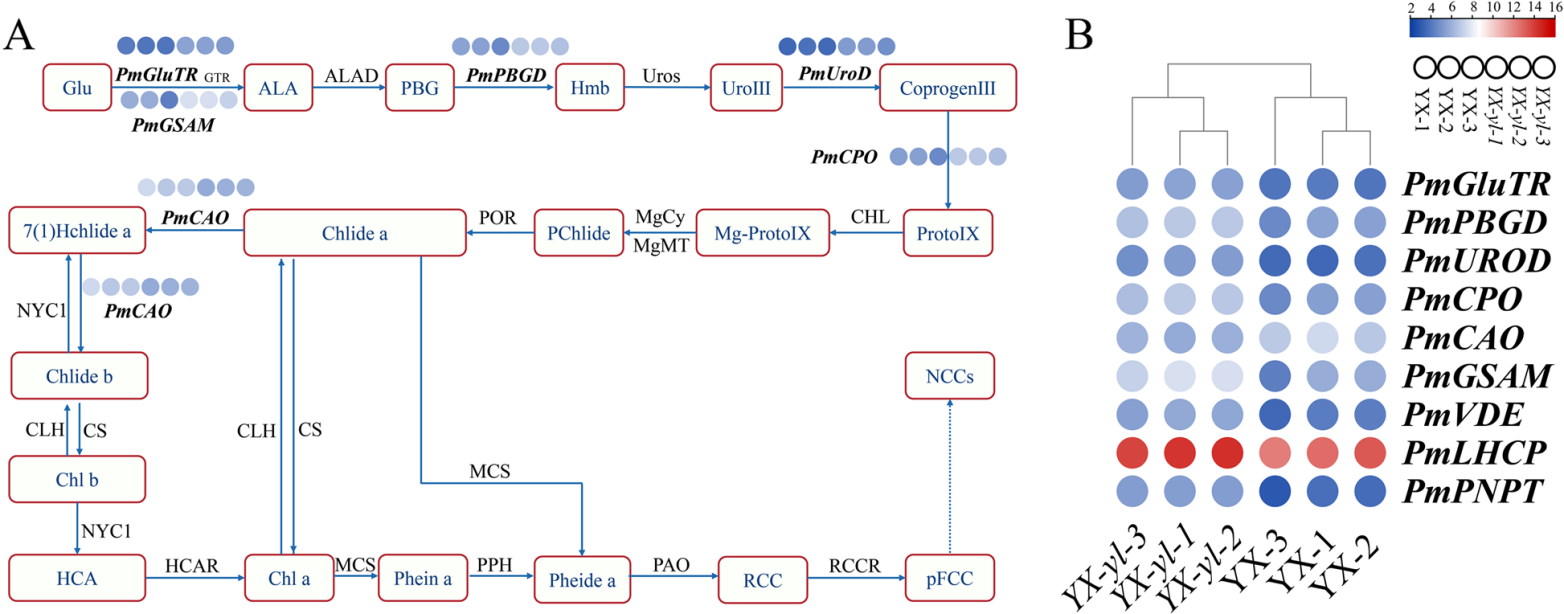
DEGs involved in the chlorophyll biosynthesis pathway. **A** Chlorophyll biosynthesis pathway. **B** Expression profile clustering of the chlorophyll biosynthesis pathway. Expression ratios are based on log_2_ fragments per kilobase of transcript per million mapped reads (FPKM) values, where each vertical solid circle represents a sample (YX-1, YX-2, and YX-3; *YX-yl-1*, *YX-yl-2*, and *YX-yl-3*), and each horizontal row represents a single gene

On the basis of the above annotations, we performed further analysis on DEGs related to chlorophyll metabolism and chloroplast development, and our resuls revealed that eight genes were upregulated, including *PmUROD*, *PmCPO*, *PmGSAM*, *PmPBGD*, *PmGluTR*, *PmLHCP*, *PmVDE*, and *PmPNPT*, whereas one gene, *PmCAO*, was downregulated (Table 3). Six genes were involved in chlorophyll biosynthesis and REDOX biological processes, and one gene (*PmVDE*) was involved in the chlorophyll metabolism pathway. Two genes, *PmLHCP* and *PmPNPT*, were involved in the regulation of chloroplast development. These results indicate that the yellow leaves have largely different metabolic activities than the wild-type leaves (Fig. 9).

**Table 3:** Function analysis of chlorophyll-related genes

Gene ID	Genes Function	log_2_FC	Up/Down
*PmCPO*	Coproporphyrinogen III oxidase activity	1.319	up
*PmUROD*	uroporphyrinogen decarboxylase activity	1.348	up
*PmGSAM*	glutamate-1-semialdehyde 2,1-aminomutase activity	1.707	up
*PmPBGD*	protoporphyrinogen IX biosynthetic process	1.258	up
*PmLHCP*	Chlorophyll A-B binding protein	1.223	up
*PmCAO*	Chlorophyllide a oxygenase	-1.165	down
*PmVDE*	violaxanthin de-epoxidase activity	1.398	up
*PmGluTR*	tRNA binding glutamate-tRNA ligase activity	1.232	up
*PmPNPT*	Probable polyribonucleotide nucleotidyltransferase 1	1.662	up

### Quantitative reverse-transcription PCR (qRT-PCR) analysis

**Fig. 10:**
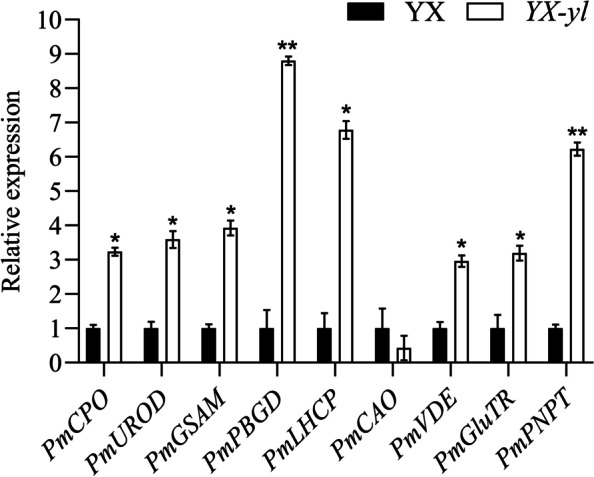
qRT-PCR of nine DEGs in *YX-yl* and YX leaves. Error bars represent ± SD. * and ** indicate significant differences (*p* < 0.05, *p* < 0.01)

To validate the reliability of DEG expression, nine DEGs were verified via qRT-PCR assay. These DEGs were involved in chlorophyll biosynthesis (*PmUROD*, *PmCPO*, *PmPBGD*, *PmGSAM*, *PmCAO*, and *PmGluTR*), chlorophyll metabolism (*PmVDE*), and chloroplast development (*PmLHCP*). The expression patterns revealed by qRT-PCR analysis were similar to those obtained by RNA-seq for the same genes (Fig. 10), indicating that the RNA-seq results of the present study were reliable for all kinds of analyses.

## Discussion

Chloroplast biogenesis, chlorophyll biosynthesis, and chlorophyll metabolism in crops are important for biomass production and economic yield. Many studies have focused on the molecular mechanisms of chlorophyll metabolism and chloroplast development in *Arabidopsis*, rice, wheat, and foxtail millet [3–9]. Leaf color mutants are ideal models for understanding photosynthetic pigment metabolism and functional chloroplast development.

Tolerance to poor-quality soil and drought as well as high photosynthetic rate are important features of broomcorn millet. To date, no leaf-color mutants or gene mapping data have been reported for broomcorn millet. Therefore, we developed a yellow-leaf mutant (*YX-yl*) with ethyl methanesulfonate treatment, and a yellow-leaf phenotype was observed during the entire growth stage. In this study, physiological and biochemical analyses integrated with transcriptome profiling were performed to elucidate the molecular mechanisms of the leaf-color mutant and explore the candidate genes controlling the mutant phenotype. The results will lay material and molecular foundations for further analysis of the photosynthetic mechanism of C_4_ plants.

### The yellow-leaf phenotype is closely associated with chlorophyll and carotenoid pigment biosynthesis

Leaf color variation is a common phenomenon in plants, and the main reason for an abnormal leaf color is an increase or decrease in chlorophyll content in the leaves. Our results revealed that the leaves of the *YX-yl* mutant exhibited much lower chlorophyll content, especially Chl b content, compared with the wild type, which played an important role in harvesting light and energy conversion. We hypothesized that the yellow phenotype of *YX-yl* leaves is caused by a deficiency in chlorophyll content, especially Chl b content, which has also been reported in cabbage [2], wheat [7], rice [6], cucumber [13], foxtail millet [8], and *Arabidopsis* [9].

Chlorophyll and carotenoid biosynthesis are determined by complex biological processes, and blockage of any step in this process can lead to a decrease in chlorophyll or carotenoid content, which in turn results in a change in leaf color. For example, the synthesis of ALA, Proto IX, and Chl a/b is regulated by glutamine-tRNA reductase, Mg-chelatase, and chlorophyllide A oxidase, respectively. If the synthesis process is inhibited or the activities of these enzymes change, we can analyze the reason for the blockage of the biosynthetic process of chlorophyll according to the accumulation of chlorophyll precursors. A previous study implied that the absence of or damage to enzymes responsible for converting Chl a into Chl b in barley might be the reason for the decrease in Chl b content [37]. Many of the reported chlorotic mutants exhibit reduced chlorophyll biosynthesis because of the lower activity of Mg-chelatase [38–40]. In the present study, comparative analysis of precursor content in the leaves of YX and *YX-yl* plants revealed that the PBG content was much higher, whereas Proto IX, Mg-Proto IX, and Pchlide were lower in *YX-yl*. We inferred that the blockage of the step between PBG and Proto IX led to a decrease in chlorophyll content, finally resulting in a yellow-leaf phenotype.

### The yellow-leaf phenotype is closely related to photosynthesis and chloroplast development

The photosynthetic rate and chloroplast ultrastructure are directly affected by leaf color mutations [4, 8, 13, 14, 41]. In a leaf-color mutant of rice, the chloroplasts are degrade, the matrix is disordered, and the thylakoids almost completely disappear [4]. In a novel leaf-color mutant of foxtail millet, disorganized thylakoids and considerably fewer starch granules have been observed [8]. Moreover, a green-yellow leaf mutant of wucai exhibited fewer chloroplasts per cell and looser stromal lamellae [41]. Similar results were found in the present study: compared with that in the wild type (YX), chloroplast development was inhibited in the *YX-yl* mutant owing to degeneration of the chloroplast ultrastructure, with fewer grana thylakoids and almost no stroma thylakoids, which might result in poor chloroplast function and accelerated leaf yellowing.

The lower content of photosynthetic pigments, combined with poorly developed chloroplasts, might be the reason for the poorer photosynthetic performance and light transfer efficiency through the photosynthetic electron transport chain. The results of both photosynthetic performance and fluorescence kinetic parameter analyses revealed that yellow color had a negative impact on the photosynthetic machinery of *YX-yl* leaves compared with the wild-type green leaves. In previous studies, a lower chlorophyll content was found to occur independently of the photosynthetic rate and light-use efficiency. For example, in foxtail millet, the leaf-color mutant maintains a relatively higher Pn and light-use efficiency [8]. Furthermore, in rice, the leaf-color mutant *ygl7* has high light energy conversion efficiency and solar energy capture efficiency [42]. The contrasting results might be due to the different gene backgrounds or different levels of chlorophyll deficiency and chloroplast development inhibition.

### The expression pattern of genes related to chlorophyll biosynthesis and chloroplast development may be the reason for the yellow-leaf phenotype

Previous results suggested that changes in genes related to chlorophyll metabolism and chloroplast development might result in a yellow-leaf phenotype. In rice, differential expression of the *OsDVR* gene, which encodes 8-vinyl reductase, between a leaf-color mutant and the wild type causes a decrease in chlorophyll content and chloroplast defect, thereby resulting in a yellow-green leaf phenotype [43]. In foxtail millet, the *SiYGL1* gene, which encodes a magnesium-chelatase D subunit (CHLD), is responsible for the mutant phenotype [8]. Our study revealed that the upregulated expression of *PmGSAM* and *PmGluTR* might be the reason for the higher PBG content. Although upregulated expression was observed for *PmPBGD*, *PmUROD*, and *PmCPO*, which encode key enzymes for chlorophyll biosynthesis, the content of Proto IX, Mg-Proto IX, and Pchlide severely decreased in the *YX-yl* leaf-color mutant, indicating that inhibition of the biosynthesis step between hydroxymethylbilane and uroporphyrinogen might be the major reason for the reduction in Proto IX and Mg-Proto IX content. Further studies should be conducted focusing on the genes or enzymes involved in this biosynthesis step. Only one downregulated gene was observed*,* namely *PmCAO*, which is involved in the biosynthesis step between Chl a and Chl b; therefore, we hypothesized that the decrease in Chl b content might be due to the downregulated expression of *PmCAO*. The *PmVDE* gene encodes a key enzyme in the lutein cycle in higher plants, which is considered the main mechanism to protect the photosynthetic apparatus from excess light energy. The development of chloroplast was inhibited in the *YX-yl* mutant, and from transcriptome analysis, we found that two genes (*PmLHCP* and *PmPNPT*) involved in chloroplast development were upregulated in the *YX-yl* mutant. These results showed that chlorophyll metabolism, chloroplast development, and chloroplast transcription were impaired in *YX-yl* plants, leading to chloroplast thylakoid degradation, reduced chlorophyll content, and yellowish leaves. Further research will be required to confirm which candidate genes play a vital role in chlorophyll metabolism or chloroplast development in *YX-yl* mutants and to explore the gene-dependent processes involved in these metabolic pathways.

## Conclusions

Phenotypic, photosynthetic, and transcriptome analyses were conducted for yellow-leaf mutant (*YX-yl)* and wild-type (YX) broomcorn millet. The yellow-leaf phenotype had lower SWPP and GWPP values, which had negative effects on grain yield. The lower Chl a and Chl b contents, abnormal chloroplast structures, and lower photosynthetic performance in the leaves of *YX-yl* indicated that chlorophyll biosynthesis (biosynthesis step between PGB and Proto IX) and chloroplast development were partially inhibited. Furthermore, we performed transcriptome analysis using RNA-seq to elucidate the molecular mechanisms underlying the yellow-leaf phenotype. We identified nine DEGs related to chlorophyll metabolism and chloroplast development. Among these genes, six genes related to chlorophyll biosynthesis, including *PmUROD*, *PmCPO*, *PmPBGD*, *PmGSAM,* and *PmGluTR*, were upregulated, whereas *PmCAO* was downregulated. In addition, *PmVDE* and *PmLHCP*, which are related to chlorophyll metabolism and chloroplast development, were upregulated in *YX-yl*. The findings of this study provide molecular evidence for the development of leaf color and chloroplasts in broomcorn millet.

## Methods

### Plant materials

The broomcorn millet yellow leaf mutant *YX-yl* was isolated from the Yi Xuan Da Hong Mei cultivar (YX), which was extensively cultivated in China. YX seeds were mutagenized with 1.0% (v/v) ethyl methanesulfonate solution. One M_2_ line, carrying the yellow-green leaf phenotype, was identified. YX (wild type) and *YX-yl* (mutant type) were used as materials for phenotypic and genetic analyses in the present study.

### Growth conditions and agronomic traits evaluated

For the YX cultivar (wild type) and *YX-yl* (mutant type), all plants were grown at the experimental station of Shanxi Agricultural University (Shanxi, China). Three uniformly developed plants were collected at the flowering stage or after harvest for phenotyping: plant height (PH), peduncle length (PL), panicle length (PaL), basal stem diameter (BSD), flag leaf length (FLL) and width (FLW), spike weight per plant (SWPP), and grain weight per plant (GWPP).

PH, PL, BSD, PaL, FLL, and FLW were measured at the flowering stage. PH was determined as the distance from the soil surface to the top of the spike. PL was measured from the base of the spike to the ligule of the flag leaf. BSD was determined as the midpoint of the basal stem from the soil surface. FLL and FLW were determined from the base to the top or midpoint of the flag leaf, respectively.

Yield components were investigated after harvest. Panicle length (PaL) was measured from the base to the top of the spike. SWPP was determined as the total spike weight per plant. The GWPP was determined as the total grain weight per plant.

### Photosynthetic pigments and chlorophyll fluorescence

The leaves of YX and *YX-yl* were collected to measure the photosynthetic pigment content at the seedling, booting, heading, and mature stages, with three biological replicates tested for each sample. The leaves were obtained from the flag leaves without the main leaf vein. Approximately 0.2 g of fresh leaves was immersed in 95% ethanol for 48 h until the leaves turned white. The absorbance of each leaf extract was measured using a UV-1800 ultraviolet/visible spectrophotometer (UV-1800pc; MAPADA, Shanghai, China) at 470, 649, and 665 nm. Chlorophyll a (Chl a), chlorophyll b (Chl b), carotenoid, and total chlorophyll content was calculated according to the following formula:

$$\mathrm{Chl a }\left(\mathrm{mg}/\mathrm{g}\right)=\left({13.95\mathrm{A}}_{665}-{6.88\mathrm{A}}_{649}\right)\times 0.01/0.2$$

$$\mathrm{Chl b }\left(\mathrm{mg}/\mathrm{g}\right)=\left({24.96\mathrm{A}}_{649}-{7.32\mathrm{A}}_{665}\right)\times 0.01/0.2$$

$$\mathrm{Car }\left(\mathrm{mg}/\mathrm{g}\right)=\left({1000\mathrm{A}}_{470}-3.27\times \mathrm{Chl a }-104\times \mathrm{Chl b}\right)\times 0.01/0.2/229$$

$$\mathrm{Chl a }\left(\mathrm{mg}/\mathrm{g}\right)=\mathrm{Chl a }+\mathrm{Chl b}$$

where Chl a is chlorophyll a content; Chl b is chlorophyll b content; Car is carotenoid content; and Chl is the total chlorophyll content.

The net photosynthetic rate (Pn), intercellular CO_2_ concentration (Ci), transpiration rate (Tr), stomatal conductance (Gs), and chlorophyll fluorescence dynamic parameters were monitored using a Li-6400 portable photosynthesis system (LI-COR, Nebraska, USA). Photosynthesis-related experiments were conducted from 08:30 to 11:00 on clear days using the flag leaf (counting from the apex) of each plant. Three individual plants were used in each experiment.

The WUE of YX and *YX-yl* was calculated using the following formula:

$$\mathrm{WUE}=\mathrm{Pn}/\mathrm{Tr}$$

### Chloroplast microstructure

Fresh leaves of YX and *YX-yl* at the fifth-leaf stage were collected, cut into 1 × 0.5 cm^2^ sections, and fixed overnight in a solution of 2.5% glutaraldehyde. The samples were washed with 0.1 M phosphate-buffered saline (pH = 7.4) three times for 15 min and then post-fixed in 1% osmium tetroxide for 2 h. The fixed leaf samples were washed with 0.1 M phosphate-buffered saline (pH = 7.4) three times for 15 min and then dehydrated in gradient ethanol solutions twice for 20 min. The dehydrated tissue was immersed in a 1:1 mixture of embedding agent-acetone for 1 h and then immersed in a 3:1 mixture of embedding agent-acetone for 3 h. Subsequently, the tissues were embedded in resin. Ultrathin sections were made from the embedded leaf samples using a Leica UC7 Ultra-wave slicer (Leica UC7; Leica, Japan), stained with uranium lead, and examined using a JEM 1230 transmission electron microscope (Jeol, Tokyo, Japan).

### Content of precursor substances for chlorophyll synthesis

The flag leaves of YX and *YX-yl* at the seedling stage were collected to analyze the content of precursor substances. Each sample contained three biological replications. ALA, PBG, Proto IX, Mg-Proto IX, and Pchlide levels were measured using a UV-1800 ultraviolet/visible spectrophotometer (UV-1800pc; MAPADA). Proto IX, Mg-Proto IX, and Pchlide content was calculated using the following formula:

$${\mathrm{C}}_{\mathrm{Mg}-\mathrm{Proto IX}}\left(\mathrm{ng}/\mathrm{g}\right)= {0.06077\mathrm{A}}_{590}-{0.01937\mathrm{A}}_{575}-{0.003423\mathrm{A}}_{628}$$

$${\mathrm{C}}_{\mathrm{Mg}-\mathrm{Proto IX}}\left(\mathrm{ng}/\mathrm{g}\right)= {0.1806\mathrm{A}}_{575}-{0.04036\mathrm{A}}_{628}-{0.04515\mathrm{A}}_{590}$$

$${\mathrm{C}}_{\mathrm{Pchlide}}\left(\mathrm{ng}/\mathrm{g}\right)= {0.03563\mathrm{A}}_{575}-{0.007225\mathrm{A}}_{590}-{0.02955\mathrm{A}}_{575}$$

### RNA extraction, library construction, and RNA-seq

Total RNA was extracted from YX and *YX-yl* leaf samples using a TRIzol RNA isolation kit (Invitrogen, USA). The purity, concentration, and integrity of the extracted RNA were assessed using a Nanodrop spectrophotometer (ND-1000; Name-drops Technologies, Wilmington, DE, USA), a Qubit 2.0 Fluorometer (Thermo Fisher Scientific, USA), and an Agilent 2100 bioanalyzer (Agilent Technologies, Santa Clara, CA, USA).

A sequencing library was prepared using the Illumina TruSeq RNA Sample PreKit. Briefly, mRNA was purified from the total RNA using poly T oligo-attached magnetic beads, fragmented, and reverse-transcribed into cDNA. Adapters were then ligated onto the cDNA molecules, and the fragments were amplified by PCR. Sequencing was performed on paired-end reads (2 × 150 bp) using an Illumina HiSeq 2500 sequencing platform. mRNA sequencing and analysis were conducted by Biomarker Biotechnology Co., Ltd. (Beijing, China).

Raw data were processed to remove low-quality reads and adapters. Next, clean data were assembled using the Trinity software (Broad Institute, Cambridge, MA, USA) [44] to obtain the unigene library of broomcorn millet. The unigene library was mapped with NR, Swiss-Prot, GO, COG, KOG, eggNOG4.5, and KEGG databases using the BLAST software [45] to obtain the annotation information of unigenes. Next, functions of unigenes were obtained using the HMMER software [46] to map unigenes with the Pfam database.

### Gene expression analysis and detection of differentially expressed genes

Clean reads were mapped with the Unigene library using the Bowtie2 software [47] and then combined with RSEM [48] for evaluation of gene expression levels. The expression level of unigenes is represented by the FPKM value [49].

Gene expression analysis was performed using the DESeq2 software [50] (www.bioconductor.org). Genes with false discovery rate (FDR) < 0.01 and fold change (FC) ≥ 2 were considered as DEGs between YX and *YX-yl*. GOseq enrichment analysis of DEGs was performed using the GOseq R package. Enrichment analysis of DEGs with annotation information between YX and *YX-yl* was conducted using the TopGO software (Version 2.18.0). Significantly enriched GO terms in DEGs were defined as GO terms with a corrected *p*-value of ≤ 0.05.

The enrichment of DEGs in KEGG pathways was assessed using the KOBAS software [51, 52], and significantly enriched KEGG pathways were defined as those with FDR ≤ 0.05.

### Validation of DEGs by qRT-PCR

To validate the sequencing data, we first selected nine DEGs for qRT-PCR. Total RNA was extracted from the leaf tissues of YX and *YX-yl*. The PrimeScript™ RT reagent Kit with gDNA Erase (Takara, Dalian, China) was used to synthesize first-strand cDNA. qRT-PCR was performed using gene-specific primers in a total volume of 10 μL comprising 5 μL of SYBR Premix Ex Taq II, 3 μL of ddH_2_O, 1 μL of primer mix (1:1 mix of forward and reverse primers at 10 μmol/μL each), and 1 μL of cDNA as a template. The reaction conditions were as follows: 30 s at 95 °C, followed by 39 cycles of 5 s at 95 °C, 30 s at the annealing temperature, and 5 s at 60 °C to 95 °C. The primers used for qRT-PCR analysis are listed in Table S6. The *actin* gene was used as an internal standard. Relative expression levels were calculated as 2^−△△ct^ [53].

## Supplementary Information

**Additional file 1.**

**Additional file 2.**

**Additional file 3.**

**Additional file 4.**

**Additional file 5.**

**Additional file 6.**

**Additional file 7.**

## Data Availability

All data generated or analyzed during this study are included in this published article and its supplementary information files. Illumina sequencing data are available at the Sequence Read Archive (SRA) under accession PRJNA839699.

## Abbreviations

Chl a: Chlorophyll a
Chl b: Chlorophyll b
PH: Plant height
PL: Peduncle length
PaL: Panicle length
BSD: Basal stem diameter
FLL: Flag leaf length
FLW: Flag leaf width
SWPP: Spike weight per plant
GWPP: Grain weight per plant
SD: Standard deviation;
Pn: Net photosynthetic rate
Ci: Intercellular CO_2_ concentration
Tr: Transpiration rate
Gs: Stomatal conductance
WUE: Water use efficiency
ALA: 5-Aminolevulinic acid
PBG: Porphobilinogen
Proto IX: Protoporphyrin IX
Mg-Proto IX: Mg-protoporphyrin IX
Pchlide: Protochlorophyllide
DEGs: Differentially expressed genes
NR: Non-redundant protein sequence database
Swiss-Prot: A manually annotated, non-redundant protein sequence database
GO: Gene Ontology
KEGG: Kyoto Encyclopedia of Genes and Genomes
COG: Clusters of Orthologous Groups of proteins
KOG: EuKaryotic Orthologous Groups of proteins
eggNOG: Evolutionary genealogy of genes, Non-supervised Orthologous Groups
qRT-PCR: Quantitative real-time polymerase chain reaction
PSII: Photosystem II
FDR: False Discovery Rate
FC: Fold change
FPKM: Fragments Per Kilobase of transcript per Million mapped reads
BP: Biological process category
CC: Cellular component process
MF: Molecular function
PhiPS2: The proportion of light quantum absorbed by PSII that is used for photochemical reactions
qN: Non-photochemical quenching coefficient
Fo: Minimal fluorescence
Fm: Maximal fluorescence under dark adaptation
Fv/Fm: Maximum energy conversion efficiency in PSII centers
qP: Photochemical quenching coefficient
ETR: Photosynthetic electron transport rate
Fv: variable fluorescence
BR: Brassinosteroid
CTK: Cytokinin
GA: Gibberellin
N50: Length of contig whose cumulative length exceeds half of the total assembled length
