# Supplementary figures and images for: Identification and function analysis of yellow-leaf mutant (YX-yl) of broomcorn millet
Source: BMC Plant Biol. 2022 Sep 27;22:463. doi: 10.1186/s12870-022-03843-y (PMC9513943; doi:10.1186/s12870-022-03843-y)

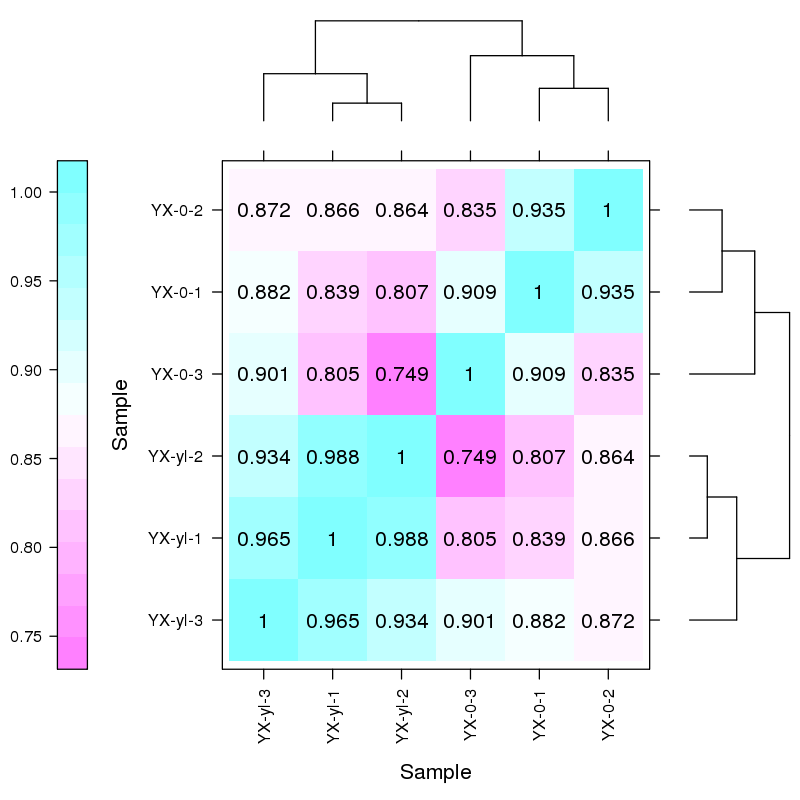

Supplement: Supplementary file 1 — Additional file 1. [file 12870_2022_3843_MOESM1_ESM.png]
